# HLA-DRB1*03 as a risk factor for microalbuminuria in same duration of type 1 diabetes: a case control study

**DOI:** 10.1186/s12882-016-0252-4

**Published:** 2016-03-31

**Authors:** Dovilė Ražanskaitė-Virbickienė, Evalda Danytė, Rimantas Žalinkevičius

**Affiliations:** Department of Endocrinology, Medical Academy, Lithuanian University of Health Sciences, A. Mickeviciaus 9, Kaunas, LT 44307 Lithuania; Institute of Endocrinology, Medical Academy, Lithuanian University of Health Sciences, Eiveniu 2, Kaunas, LT 50009 Lithuania

**Keywords:** Type 1 diabetes mellitus, Albuminuria, AER, HLA, DR3, DR4

## Abstract

**Background:**

Increased urinary albumin excretion rate is the earliest clinical manifestation of diabetic nephropathy. The development of microalbuminuria in patients with type 1 diabetes mellitus (T1D) usually begins 5 to 15 years after the onset of diabetes. The rate of progression of diabetic nephropathy varies considerably among patients and not always can be explained solely by glycaemic control. The evidence suggests that genetic susceptibility may play a role in the development of diabetes microvascular complications, besides the presence of such risk factors as hyperglycaemia, hypertension, dyslipidaemia and smoking. The aim of the study was to evaluate a link between known genetic risk factors for type 1 diabetes mellitus (HLA-DR3/DR4) and microalbuminuria among patients with the same durations of diabetes.

**Methods:**

Ninety-nine patients with T1D at the age 18–35 years were recruited for the study. The urine albumin excretion rate was normal when <30 mg/24 h; microalbuminuria 30–300 mg/24 h. Genotypes were investigated in 39 patients with normal albumin excretion rate and duration of diabetes 13.46 ± 3.72 years and in 60 patients with microalbuminuria and duration of diabetes 15.28 ± 4.08 years (*p* = 0.11). Genetic typing of DR3 and DR4 antigens successfully was performed for 99 subjects. Statistical analysis was performed using SPSS v. 20.0.

**Results:**

Genotyping of 99 patients with T1D was performed: no DR3 and DR4 risk alleles were found in 22 (22.22 %) cases, DR3 alleles were present in 47 (47.48 %) cases, DR4 alleles in 25 (25.25 %) cases, and DR3/DR4 alleles in 5 (5.05 %) cases. The highest 24 h albumin excretion rate was found in patients with DRB1 gene expressed DR3 risk alleles group, the lowest - in patients with DRB1 gene with no expression of both DR3 and DR4 antigen. We confirmed the 1.87 (*p* = 0.021) increased relative risk for microalbuminuria in patients with DR3/DR3 alleles and same duration of diabetes. The distribution of DR3 and DR4 risk alleles was not associated with cardiovascular autonomic neuropathy both in patients with normal albumin excretion rate and microalbuminuria (1.6 vs 2.1; *p* = 0.21).

**Conclusions:**

The 1.87 (*p* = 0.021) increased relative risk for microalbuminuria was found in patients with DR3/DR3 alleles and the same duration of diabetes.

## Background

Type 1 diabetes is a complex disease, the course of which is influenced by many interacting genetic and environmental factors [1]. Glycaemic control and diabetes duration are the most important risk factors for the development of late diabetic complications. However, the rate of progression of nephropathy, retinopathy and neuropathy varies considerably among patients and not always can be explained solely by glycaemic control. Diabetic nephropathy is one of the most threatening complications of T1D, and its rate and severity directly correlates with the duration of T1D [2]. The presence of diabetic nephropathy is related with the greatest risk of premature death in patients with T1D [3]. Patients with diabetic nephropathy are likely to have neurological complications of diabetes including cardiovascular autonomic neuropathy (CAN), the most often overlooked serious complication of diabetes [4]. Therefore, it is very important to detect kidney damage early and to stabilize effectively, because it is reversible in the initial stages [5]. Microalbuminuria is an early sign of diabetic nephropathy. The development of microalbuminuria in patients with T1D usually begins 5 to 15 years after the onset of diabetes and then increases over time, a small proportion of patients develop microalbuminuria within less than 5 years of disease onset [6, 7]. Clinical nephropathy usually occurs after 15–25 years diabetes duration and rarely newly develops when T1D duration is more than 30 years [8].

The mechanisms of development of diabetic nephropathy are not fully clear. The evidence suggests that genetic susceptibility may play a major role in the development of microvascular complications, besides the presence of such risk factors as hyperglycaemia, hypertension, dyslipidaemia and smoking. Based on underlying pathogenesis, polymorphisms of several candidate genes belonging to multiple pathways have been investigated, like the genes related to mechanisms of hyperglycaemia-induced damage (such as advanced glycation end-products and reactive oxygen species increased formation, augmented activity of the aldose reductase pathway); genes related to the renin-angiotensin system; genes coding for cytokines, growth factors and its receptors, glucose transporters; among many others [9].

The Major Histocompatibility Complex (MHC) region on chromosome 6p21 has been robustly replicated as a major risk locus in many immune-mediated diseases, including T1D. Certain haplotypes of human leukocyte antigen (HLA) genes are known to confer the main genetic risk or protection within the MHC region [1]. The major T1D susceptibility locus maps to the class II loci HLA-DRB1 and HLA-DQB1 on chromosome 6p21. The highest risk DR/DQ haplotypes for T1D are DR3-DQA1*0501-DQB1*0201 (DR3) and DR4-DQA1*0301-DQB1*0302 (DR4). Both DR3and DR4 are very strongly associated with T1D: over 93 % of T1D patients have at least one of these alleles, compared to 43 % of controls [10]. Other non-HLA T1D loci in combination have smaller effects on disease risk compared to HLA [11].

The aim of the study was to evaluate a link between known genetic risk factors for type 1 diabetes mellitus (HLA-DR3/DR4) and microalbuminuria.

## Methods

Ninety-nine patients with T1D at the age 18–35 years were recruited for the case–control study. The following standard-case definition criteria for T1D were used: diagnosed as diabetic patient by physician; placed on insulin therapy before the 30th birthday; resident of his/her country at the time of the first insulin administration [12]. The anamnesis of microalbuminuria, hypertension renal failure or consumption of such drugs like ACE (angiotensin-converting-enzyme) inhibitors or ARB (aldosterone receptor blockers) was the exclusion criteria in our study. The information about patients was received from endocrinologists and general practitioners of Kaunas County.

The evaluation 24 h urine albumin excretion rate (AER) was based on C. Mogensen [13]: normal AER <30 mg/24 h; microalbuminuria - AER 30–300 mg/24 h, macroalbuminuria - AER >300 mg/24 h. Subjects with macroalbuminuria, - AER >300 mg/24 h were excluded from investigation. Genotypes were investigated in 39 patients with normal AER and duration of T1D 13.46 ± 3.72 years (cases) and in 60 patients with microalbuminuria and duration of diabetes 15.28 ± 4.08 years (controls) (*p* = 0.11). Genetic typing of DR3 and DR4 antigens was performed for all 99 subjects. The diagnosis of CAN was based on the results of a battery of autonomic tests (heart rate at rest, heart rate response to deep breathing, standing up, systolic blood pressure response to standing up and diastolic blood pressure response to isometric exercise). The diagnosis of CAN was confirmed when 2 or more pathological tests were present. Standardised questionnaires were completed to obtain information on demographic data, clinical events, medications and life style.

The study protocol was approved by the local Ethics committee of Lithuanian University of Health Sciences (Kaunas city); all participants provided written informed consent.

### Methods of genotyping

DNA was extracted from peripheral blood leukocytes by salt precipitation. The isolated DNA was dissolved in sterile 1xTE buffer. Isolated DNA concentration was measured with a spectrophotometer, and purity was assessed by measuring the optical density.

Investigated DNA fragment was amplified with PCR reaction using 100 ng of subjects DNA, dNTP mix, Taq polymerase, 1.25 Dream U and reaction buffer. Specific primers were used for amplification of 213 bp (base pairs) PCR fragment size, which create an artificial restriction fragment length polymorphism in DRB1 gene - DR3 RFLP: 5′ CCG CTG CAC TGT GAA GCT CTC CAC AAC CCC GTA GTT GTG TCT GCA CTAG 3′ and DR4.04: 5′ CGG GTG CGG TTC CTG GAC AGA TAC TTC GAT 3′. PCR was performed using gradient Eppendorf firm thermal cycler. PCR reaction products were visualised on 1.5 % agarose gel. Restriction reaction was performed using 5 units of restriction endonuclease Spe 1 (Enzyme) and 1 Dde (Enzyme) in 15 μl of reaction mixture (composition - 5 μl of the PCR product, restriction endonuclease Spe 1 and 0.5 μl Dde, 1.5 ul 10× reaction buffer and 7.5 μl of water). After 2 h Sau 3A1 enzyme, which recognizes GATC sequence in DR4 allele was added and a fragment of 186 bp is obtained.

Restriction of ACTAGT sequence, which presents only in DR3 or DRB7 allele, was recognized by restriction of endonuclease Spe 1 and obtained fragment of 164 bp. Dde 1 allows to distinguish between DR3 or DRB7 alleles because it recognizes two places of CTNAG in DRB7 allele (codons 51–52 and 58–59), and neither one place in DR3 allele. If it is DRB7 allele Dde 1 cuts PCR into three fragments of 106, 86, and 21 base pairs. If Dde cutting site is located on another allele, there is no the second cutting site, and are presented 106/107 bases duplets.

Restriction fragment size was evaluated in 4 % agarose gel after electrophoresis and colouring EtBr. Gel was documented and restriction fragment sizes were assessed using BioRad gel documentation system.

The assessment of HLA genotyping results:

- N/N – DRB1 gene does not express neither DR3 nor DR4 antigen (both alleles determine the absence of DR3 and DR4 antigen - homozygote);
- DR3/DR3 – subject has DR3 antigen (both alleles determine DR3 antigen- homozygote);
- DR4/DR4 – subject has DR4 antigen (both alleles determine DR4 antigen- homozygous);
- DR3/DR4 – subject has DR3 and DR4 antigens (heterozygote);
- DR3/N – subject has DR3 antigen (heterozygote);
- DR4/N – subject has DR4 antigen (heterozygote).

### Statistical analysis

Statistical analysis was performed using SPSS v. 20.0 (License No. 9582494). The sample size was calculated during a pilot study using PASS (Power analysis and sample size software). The power of the study was selected to be β = 0.8, and the confidence level - α = 0.05. Data are presented in absolute value and frequency. Data are presented as means and standard deviations. Used statistical methods: variables of two independent samples were compared using a Mann–Whitney test. Chi-square (*χ*2) was used for rates comparison. Risk alleles search was performed using univariate odds ratio analysis. Results were interpreted as statistically significant at an error probability of *p* <0.05.

## Results

The 99 patients with T1D (47 males and 52 females) were included in the study. Genotypes in two homogeneous groups according to the age of T1D diagnosis, duration of diabetes, gender and HbA1c were analysed. The characteristics of patients are summarized by AER status in Table 1. The proportion of CAN was significantly greater in case group (6.81 %) as compared with control group (2.32 %) (*p* = 0.03).

**Table 1:** Characteristics of the study subjects according to AER

	Normal AER (control patients)	30 < AER <300 mg/24 h (case patients)	2- tailed *p*
*n*	39	60	
Age at diagnosis (years)	12.25 ± 6.12	13.61 ± 5.41	0.26
Age at entry (years)	26.53 ± 7.89	29.37 ± 5.56	0.19
Duration of diabetes (years)	13.46 ± 3.72	15.28 ± 4.08	0.11
Gender (M/F)	18/21	29/31	0.31
HbA_1c_ (%)	7.8	8.0	0.43
Systolic blood pressure (mmHg)	123 ± 11	125 ± 13	0.38
Diastolic blood pressure (mmHg)	75 ± 8	78 ± 10	0.40
CAN (%)	2.32	6.81	0.03

The highest 24 h AER was found in patients with DRB1 gene expressed DR3 risk alleles group. The lowest AER was found in patients with DRB1 gene with no expression of both DR3 and DR4 antigen (Table 2). Assessing DRB1 gene’s N/N, DR3 and DR4 risk alleles combinations and microalbuminuria, the greatest significance was achieved between N/N and DR3 alleles groups.

**Table 2:** Mean AER (mg) and ±95 % confidence intervals associated with combinations of alleles

Alelles	Mean	±95 % CI
N/N	23.95	13.57–34.33
DR3/DR3+N/DR3	53.00	23.83–82.17
DR4/DR4+N/DR4	39.70	20.96–58.45
DR3/DR4	30.00	18.88–78.88

Note: *CI* confidence intervals

Genotyping of 99 patients with T1D was performed: no DR3 and DR4 risk alleles were found in 22 (22.22 %) patients, DR3 alleles were present in 47 (47.48 %), DR4 alleles in 25 (25.25 %), and DR3/DR4 alleles in 5 (5.05 %) (Table 3). According to the frequency of different combinations of alleles we didn’t find statistical difference among case (AER 30–300 mg/24 h) and control (AER <30 mg/24 h) groups.

**Table 3:** Frequency of different combinations of alleles in normal AER and microalbuminuria

	N/N	DR3/DR3+N/DR3	DR4/DR4+N/DR4	DR3/DR4
	*n* (%)	*n* (%)	*n* (%)	*n* (%)
Normal AER (control patients)	10 (25.64)	16 (41.03) ^a^	12 (30.77)	1 (2.56)
30 < AER <300 mg/24 h (case patients)	12 (20.00)	31 (51.67) ^a^	13 (21.67)	4 (6.67)
2-tailed p	0.44	0.06	0.19	–

Note: ^a^N/N vs DR3/DR3+N/DR3, DR4/DR4+N/DR4, DR3/DR4

We compared by DRB1 gene’s N/N, DR3 and DR4 risk alleles in patients with T1D, most case patients had DR3 risk alleles: 31 (51.67 %) patient had DR3 alleles, 13 (21.67 %) patients had DR4 alleles and 4 (6.67 %) patients had DR3/DR4 alleles. We confirmed the 1.87 (*p* = 0.021) increased relative risk for microalbuminuria in patients with DR3/DR3 alleles and the same duration of diabetes (Table 4).

**Table 4:** Univariate analysis of risk of microalbuminuria according genotypes

Genotype	OR	±95 % CI	2-tailed p
N/N	0.62	0.43–0.91	0.392
N/DR3	0.73	0.23–0.87	0.411
N/DR4	0.31	0.24–1.89	0.371
DR3/DR3	1.87	1.21–3.16	0.021
DR4/DR4	0.87	0.67–1.23	0.423
DR3/DR4	–	–	–

Note: DR3/DR4 - the number of cases in groups 4/1, *OR* odds ratio, *CI* confidence intervals

The distribution of DR3 and DR4 risk alleles was not associated with CAN both in patients with normal AER and microalbuminuria (1.6 vs 2.1; *p* = 0.21).

## Discussion

Microalbuminuria is an early sign of diabetic nephropathy. Microalbuminuria is seldom found in patients within first 5 years from onset of T1D, usually microalbuminuria starts at 5 to 15 years of duration of diabetes and then increases over time [6, 7]. In a systematic review of nine longitudinal studies examining moderately increased albuminuria in 7938 patients with T1D, the overall prevalence of moderately increased albuminuria was 28 % at a mean duration of T1D of 15 years [14].

J.H. Warram and co-authors state that microalbuminuria can be found in about 20 % of patients with type 1 diabetes with duration of disease 20 years and in a 50 % with duration of disease more than 30 years [7]. For kids and teens advanced stage of diabetic nephropathy is seldom found [15]. Several studies describe the rapid clinical and histological development of diabetic nephropathy in children and adolescents at 4–11 years duration of disease [16, 17]. Familial tendency to develop this diabetes complication was determined [18], showing the importance of genetic factors. But still no individual gene that results in kidney damage in patients with type 1 diabetes was identified.

The impact of apolipoprotein E gene [19], growth arrest-specific 6 gene (Gas6) and their receptors Ax1 [20], polymorphism of the enzyme V16A of manganese superoxide dismutase [21], insulin gene [22], DQA1 and DQB1 haplotypes [23], HLA-DR3 and DR4 alleles [24] are investigated to clarify the reasons for the development of diabetic nephropathy. HLA has a significant impact not only to the development of T1D, but also to diabetes complications. The aim of the study was to evaluate a link between HLA-DR3/DR4 and microalbuminuria among patients with the same durations of diabetes.

The highest risk DR/DQ haplotypes for T1D are DR3-DQA1*0501-DQB1*0201 (DR3) and DR4-DQA1*0301-DQB1*0302 (DR4), and these alleles account for 30–50 % of genetic T1D risk [10]. Although 40 % of whites in the US have an HLA-DR3 or -DR4 allele, at least 1 of these alleles is present in 95 % of patients with T1D. The estimated risk of developing T1D for the general population in children who have the HLA-DR3/4 genotype is approximately 1 in 15 to 1 in 25 vs. a risk of 1 in 300 in the general population [25]. Only 2.4 % of the general population carries this genotype compared to 30–40 % of T1D patients. Although this genotype confers extremely high risk, there is a spectrum of risk associated with HLA DR/DQ genotypes—from increased, to neutral, to protective [26].

Very early small studies looked at HLA in people with diabetic nephropathy, but no reliable associations was found [27–29]. The assumption that the HLA DRB1*04 alleles may protect against development of diabetic nephropathy appeared recently. A. Svejgaard et al. investigated 71 patients with long duration of T1D, 24 had diabetic nephropathy and 51 normal AER, and found a marked protective effect of DR4 allele (*p* = 0.02) [30]. In our study we have found the highest risk of increased AER in T1D patients with DRB1 gene expressed DR3 risk allele. Also the lowest AER we found in patients with neither DR3 nor DR4 antigen expressed. Genetics of Kidneys in Diabetes (GoKinD) study [31], examined patients with T1D with nephropathy (*n* = 829) and without nephropathy (*n* = 904). Results of this study showed, that diabetic probands who were homozygous for HLA DRB1*04 were 50 % less likely to have nephropathy than probands without any DRB1*04 alleles. In heterozygous carriers, a protective effect of this allele was not as clearly evident. This association was seen in probands with both short (<28 years, *p* = 0.02) and long (≥28 years, *p* = 0.0001) duration of diabetes. Interestingly, DRB1*04 appears to be both a risk allele for T1D and a protective allele for nephropathy. In our study patients who were homozygous for HLA DRB1*04 were 50 % less likely to have microalbuminuria than patients who were homozygous for HLA DRB1*03 allele. The main weakness of our study is the small sample size. The power of the study was selected to be β = 0.8, and the confidence level - α = 0.05. We calculated that we need to find 334 patients (case and controls). Finally we could include and analyze only 99 patients, so further research is necessary to confirm our results. The other limitation is that a population of our study was only caucasians, and possibly our findings couldn’t be applied to other races.

## Conclusions

The 1.87 (*p* = 0.021) increased relative risk for microalbuminuria was found in patients with DR3/DR3 alleles and the same duration of diabetes.

### Availability of data and materials

Data supporting our findings can be found at http://www.lsmuni.lt/en/library/about-library-and-information-centre/, http://www.kmu.lt/endokrinologija/index.php?psl=pradzia and http://vddb.library.lt/obj/LT-eLABa-0001:E.02~2011~D_20110922_122348-00650.

## Abbreviations

AER: albumin excretion rate
CAN: cardiovascular autonomic neuropathy
HLA: human leukocyte antigen
MHC: the Major Histocompatibility Complex
T1D: type 1 diabetes
